# The treatment of the atrophic clavicular nonunion by double-plate fixation with autogenous cancellous bone graft: a prospective study

**DOI:** 10.1186/s13018-020-02154-y

**Published:** 2021-01-07

**Authors:** Jun Zhang, Peng Yin, Bo Han, Jianmin Zhao, Bo Yin

**Affiliations:** 1 Department of Orthopaedics, The affiliated Hospital of Inner Mongolia Medical University, Hohhot, 010010 China; 2 Department of Orthopaedics, Beijing Chao-Yang Hospital, China Capital Medical University, Beijing, 100020 China

**Keywords:** Ununited fractures, Clavicle, Internal fixators, Bone transplantation

## Abstract

**Background:**

The objective of this study is to assess prospectively the effectiveness of double-plate fixation combined with autogenous cancellous bone graft in the treatment for the atrophic clavicular nonunion.

**Methods:**

Between February 2013 and November 2017, a total of 12 patients with atrophic clavicular nonunion (mean age, 40.4 ± 9.0 years, range, 27–60 years) were treated by double-plate fixation with autogenous cancellous bone graft in our institute. The Constant Score System was used to evaluate the preoperative and final outcomes. The Short Form-36 (SF-36) outcome questionnaire was used to assess the final clinical results.

**Results:**

All patients were followed-up, with the average follow-up of 34.7 ± 6.7 months (range, 24–48 months). The healing rate was 100% in our study. The mean time of bony union was 9 weeks (range, 8–10 weeks). One patient had a postoperative superficial infection, and the patient was cured by oral antibiotics and wound dressing. No implant-related complications (plate-screw loosening or breakage) were observed postoperatively. No vascular injury, neural impairment, or thoracic outlet syndrome was discovered preoperatively or postoperatively. There is a statistical significance between the preoperative and the postoperative constant scores (*P* < 0.05). All the patients were satisfied with their final clinical results by SF-36 outcome questionnaire. Average scores of the physical function and bodily pain components of the SF-36 were 94.2 ± 7.3 and 92.5 ± 5.8, respectively.

**Conclusion:**

Our results presented that double-plate fixation with autogenous cancellous bone graft is an effective treatment for atrophic clavicular nonunion, especially for those with a significant bone defect.

## Background

Fractures of the clavicle account for 2.6–10% of all fractures [1]. Most clavicle fractures could be cured by conservative treatment, except long-term immobilization-caused various complications, such as pain, deformity, and functional disorder of the shoulder [2]. Therefore, most patients choose surgical treatment to achieve quicker recovery of function and better alignment, especially for the patients with a comminuted clavicle fracture [2, 3]. However, surgery-related complications still exist, and clavicle nonunion is one of the main complications. Olsen BS reported that the rate of clavicle nonunion after conservative treatment was between 0.1% and 5% [4], and 2.6% after surgical treatment [5].

Many surgical techniques have been described for the treatment of clavicle nonunion, including intramedullary fixation with long pins and screws [6], vascularized fibula [7], Ilizarov External Fixator [8], lag screws fixation [9], and bone grafting [4, 10]; however, there still a different consensus on the best treatment, and every solution has its own set of disadvantages. For instance, the Ilizarov technique has been successfully applied in the treatment for clavicle nonunion, but it always causes pin tract infections and subjective discomfort [8]. Although intramedullary fixation with long pins has partly improved the stability of the nonunion site, many complications still exist, such as migration of the pins and pin breakage. In addition, implantation of the intramedullary pins is difficult because of the irregular anatomic morphology of the clavicle [11–14]. Therefore, more effort is needed to obtain an optimal operative treatment for clavicle nonunion.

The cardinal surgical treatment for a clavicle fracture is one-plate fixation [15]. However, the stability of clavicle nonunion treated with a single plate is unstable in some special cases, especially atrophic nonunion with a bone defect. Therefore, we attempted to use a double-plate construct (an AO reconstruction plate or a LC-DCP plate with auxiliary minor reconstructive plate) to provide a rigid mechanical environment for the treatment of the atrophic clavicular nonunion; in addition, an autogenous cancellous bone was also used for improving the biological environment at the site of nonunion. In this study, we assessed prospectively the effectiveness of the treatment of the atrophic clavicular nonunion by double plate fixation with autogenous cancellous bone graft and aim to provide an alternative surgical method for the disease.

## Methods

**Fig.1:**
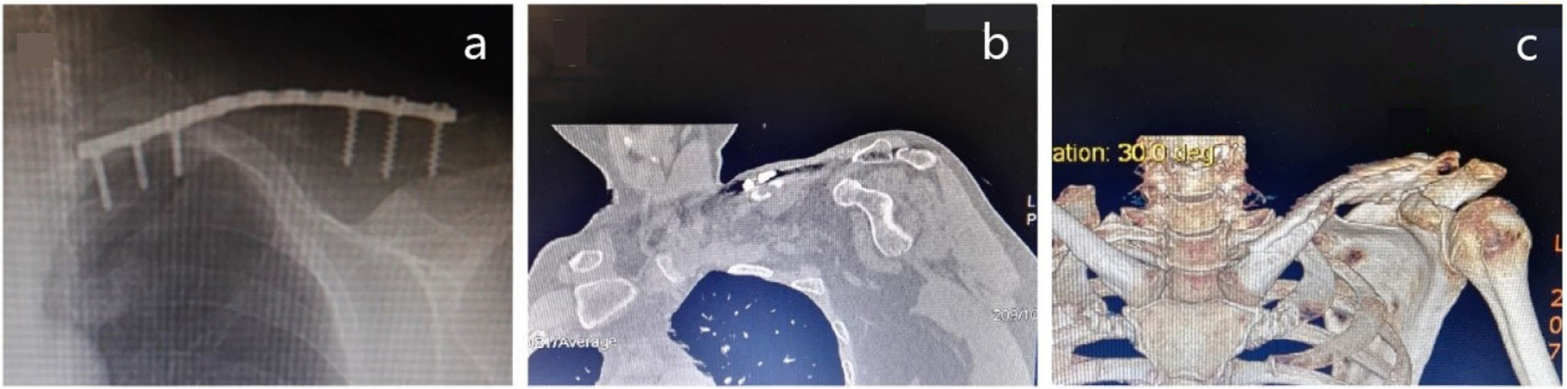
**a** Clavicle nonunion with plate fixation at 9 months. **b** Nonunion confirmed by CT scan. **c** Nonunion was showed in the 3D reconstruction of the clavicle

We analyzed all patients with clavicle nonunion after operative treatment from February 2013 to November 2017 retrospectively. Our inclusion criteria were adult atrophic clavicular nonunion, and the exclusion criteria were (1) infectious nonunion, (2) pathological nonunion, and (3) nonunion after expectant treatment. According to the inclusion and exclusion criteria, 12 cases were enrolled. Series of laboratory examinations were performed to exclude infection, including C-reactive protein level, white blood cell count, and erythrocyte sedimentation rate. The cutaneous temperature was also measured. A pathological examination of the removed nonunion bone tissue during the revision was carried out to confirm the aseptic bone nonunion. The bone nonunion was defined according to the American Food and Drug Administration (FDA) standard. The nonunion is defined as a fracture that did not heal for 9 months and had no tendency to heal for 3 months [16, 17]. Before the operation, atrophic sclerosis of the fracture end and sealing of the medullary cavity were identified in X-section. In some patients, we determine nonunion by CT scan (Fig. 1).

In this study, 12 patients with atrophic nonunion were included in the study. Five patients had a right/dominant clavicular nonunion, whereas the remaining seven patients had a left clavicular nonunion. The mean age is 40.4 ± 9.04 years (range, 27–60 years). More details were listed in Table 1.

**Table 1:** Demographic characteristics and clinical outcomes of the patients

Patient no.	Gender/age (years)	Non-union type	Fracture type AO/OTA classification	Operative time (minutes)	Time to union (weeks)	Complication	Follow-up (months)	Preoperative constant score	Final outcome constant score
1	M/37	Atrophic	B3	95	9	None	48	22	79
2	M/39	Atrophic	B2	90	9	None	42	26	88
3	M/60	Atrophic	B1	80	10	infection	40	18	70
4	F/38	Atrophic	B2	105	9	None	38	23	80
5	M/48	Atrophic	B2	85	8	None	37	32	82
6	M/46	Atrophic	B1	100	9	None	35	40	89
7	M/45	Atrophic	B1	95	9	None	33	19	78
8	M/27	Atrophic	B1	90	8	None	32	35	88
9	F/42	Atrophic	B2	85	9	None	30	35	82
10	F/29	Atrophic	B2	90	8	None	29	38	80
11	M/32	Atrophic	B3	90	9	None	28	40	85
12	M/42	Atrophic	B3	100	9	None	24	33	84

The patients were placed supine in a beach chair position. The anesthesiologist gave the patient brachial plexus and cervical plexus anesthesia. The longitudinal incision was made along the inferior aspect of the clavicle overlying the nonunion site. After arriving at the nonunion site, sclerotic bones were debrided until the occurrence of the paprika sign (paprika sign is bleeding from the broken ends of the nonunion). It is used to determine the blood flow at the fracture end and assess the viability of the bone fragment) [18], and then, the K-wire was used to drill the medullary cavity. The cardinal plate above the clavicle was usually 9 hole AO locking reconstruction plate or LC-DCP plate (3.5 mm, 6 screws, double cortex fixation). Plates are contoured to the patient’s clavicular anatomy. We used 360° sufficiently surrounding bone defect site autogenous bone graft technique. The bone autograft material was acquired from the anterior iliac crest. The auxiliary plate in the anterior-posterior plane is usually a 7–9 hole general reconstruction plate or AO LCP(2.7 mm, 4 screws, single cortex fixation). After satisfactory X-ray examination, a large amount of normal saline was used to rinse, a drainage tube was placed, then fascia, subcutaneous tissues, and superficial skin were sutured layer by layer.

Two weeks after surgery, patients were allowed to use the arm out of the sling with the assistance of a physiotherapist. Three weeks later, the patients can resume gentle exercises. Most of the patients were able to elevate the arm for above-shoulder activities at 3 weeks after the operation. Four weeks after surgery, the patients can return to weightlifting and full exercises. The final clinical results were evaluated by the constant score system and Short Form-36 (SF-36) outcome questionnaire [19, 20]. The constant score could provide a so-called numerical dual assessment: the objective evaluation index including shoulder mobility and muscle strength (65 points) and the subjective evaluation index including pain and functional activity (35 points). It includes the presence of pain (15 points), functional activities (20 points), shoulder mobility (40 points), and muscle strength (25 points) [21]. A score of 100 indicates excellent shoulder function.

The data were analyzed by SPSS 19 software (IBM, Armonk, NY, USA) and the associated IRR package. In this study, the statistical analysis was carried out including the arithmetical mean, standard deviation, and 95% confidence interval of demographic characteristics and clinical outcomes of the patients. The statistical significance was analyzed for the difference between mean pre- and postoperative constant scores using Student’s *t* test for small, dependent samples.

A value of *P* < 0.05 was considered to indicate statistical significance.

## Results

The mean operation time was 85.8 min (range, 80–105 min), the mean intraoperative bleeding volume was 220 ml (range, 150–280 ml), and the mean hospitalization was 4 days (range, 3–7 days).

**Fig. 2:**
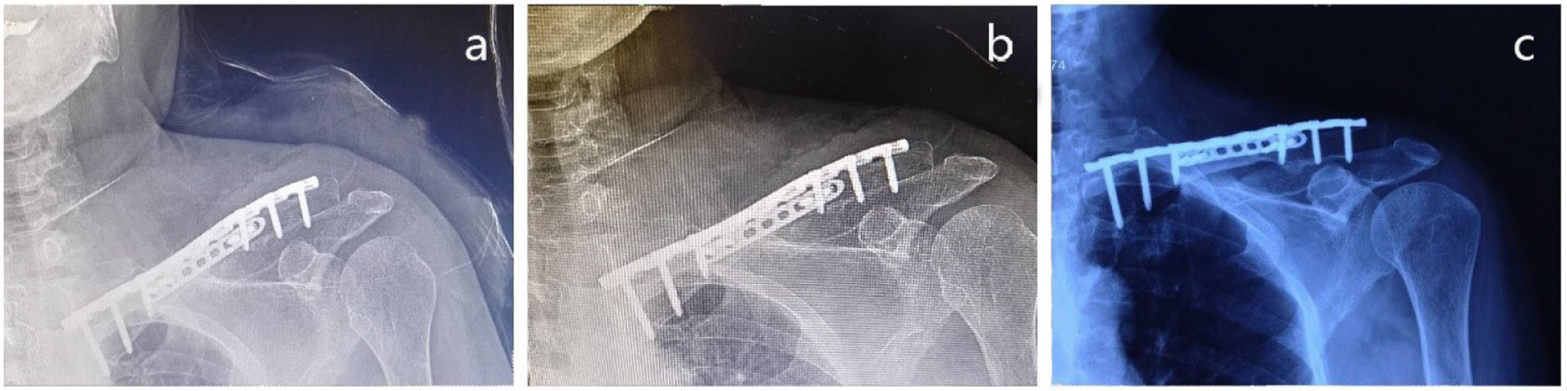
**a** 2-Plate revision postoperative immediately. **b** 2-Plate revision postoperative 3 months. **c** 2-Plate revision postoperative 1 year

**Fig. 3:**
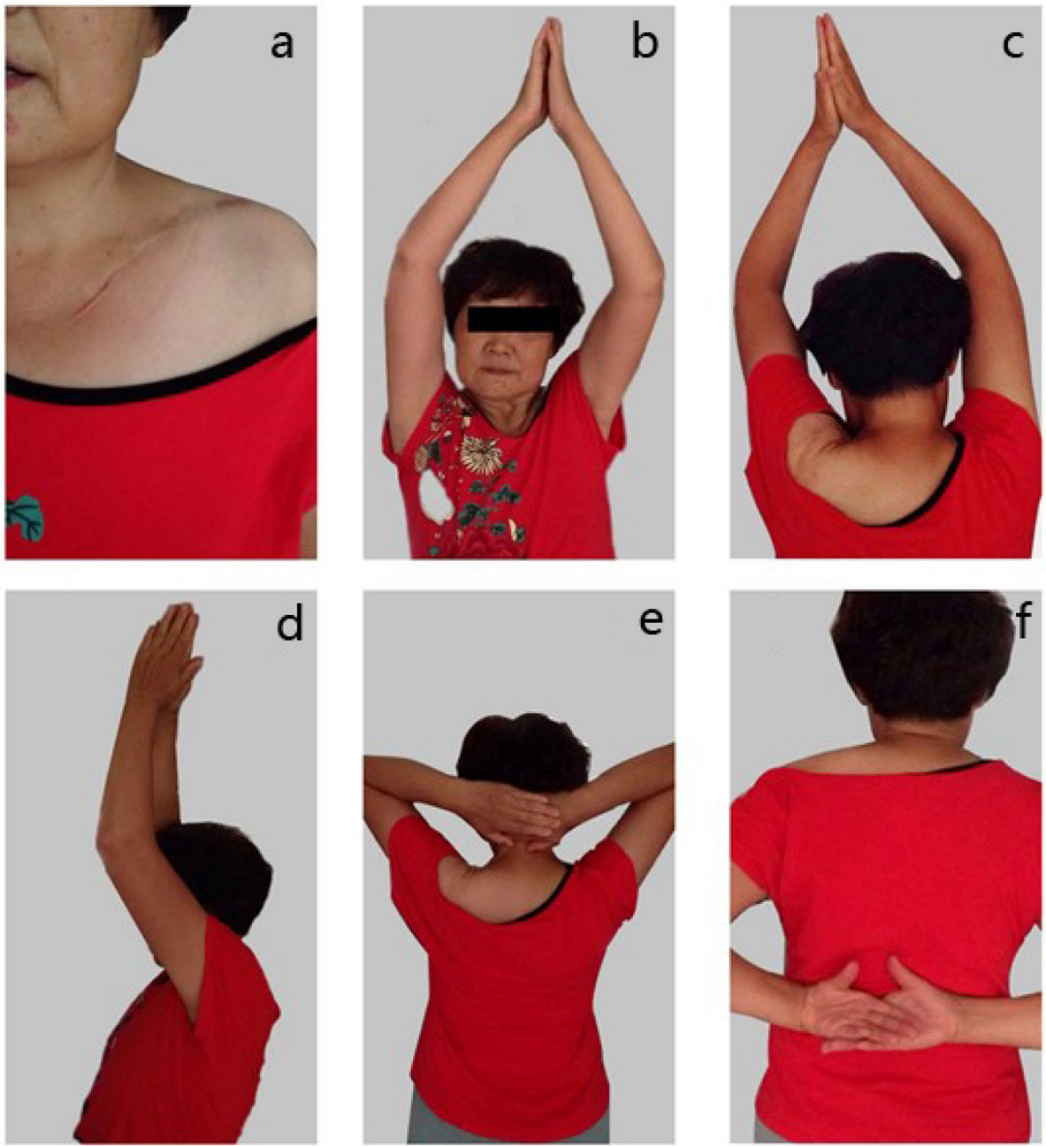
**a** The appearance of skin incision and shoulder function after operation. **b**–**f** Shoulder function after the operation

The follow-up for all patients is complete. The average time of follow-up is 34.7 ± 6.7 months (range, 24–48 months). The mean time of bone union was 9 weeks (range, 8–10 weeks) (Fig. 2). There was a statistical significance between the preoperative and the postoperative constant scores (*P* < 0.05). The preoperative and postoperative constant scores were shown in Table 1. Average scores noted on the physical function and bodily pain components of the SF-36 were 94.2 (range, 92–99) and 92.5 (range, 90–99), respectively (Fig. 3). The average scores of the constant score and SF-36 outcomes questionnaire on the patients were listed in Table 2.

**Table 2:** Average scores of the constant score and SF-36 score system and SF-36 outcomes questionnaire

The Constant score		SF-36 score	
Scale	Score	Scale	Score
Pain Functional activities Shoulder mobility Muscle strength Total score	11.0 ± 2.6 17.0 ± 6.5 35.2 ± 1.8 20.3 ± 5.3 77.1 ± 17.5	Physical function Bodily pain Role-physical General health Vitality Social function Role-emotional Mental health	94.2 ± 7.3 92.5 ± 5.8 95.8 ± 0.8 91.7 ± 2.5 96.3 ± 6.1 97.2 ± 3.7 94.1 ± 2.5 96.5 ± 4.4

The healing rate is 100% in our study. One patient had a postoperative superficial infection, and the patient was cured by oral antibiotics and wound dressing. No implant-related complications (plate-screw loosening or breakage) were noted postoperatively. No vascular injury, neural impairment, or thoracic outlet syndrome was observed preoperatively or postoperatively. So far, all patients did not require the removal of the internal fixation.

## Discussion

This is a prospective study about the treatment of the atrophic clavicular nonunion by double-plate fixation with autogenous cancellous bone graft. The present study shows that the operation acquired satisfying outcomes. The healing rate is 100% in our study. Only one patient had a postoperative superficial infection, which was cured by oral antibiotics and wound dressing. No distinct complications emerged postoperatively.

In our study, the success rate (100% union rate) and mean time of union (9 weeks) are comparable with the previous results published by Schnetzke et al. [22], Hui-Kuang Huang et al. [23], Slavko et al. [8], Kabak et al. [24], Jones et al. [25], and Sadiq et al. [26] (Table 3). Sadiq et al. reported that the incidence of clavicular nonunion was 3%. Five patients were treated with double plate, and the final healing rate was 100% [26]. The double-plate fixation technique could achieve a higher healing rate. DPF is more effective to accelerate fracture healing, by providing a more stable mechanical environment in fracture healing. Of course, major fixation stability was realized by locking reconstruction or LC-DCP plate above the clavicle. Firstly, the superior plate could provide the main strength of the structure of double plates. Secondly, the anterior-posterior plate could immobilize bone defects and neutralize the distribution of the part of stress. Moreover, it also could avoid minor displacement of the anterior-posterior plate and strengthen the antirotation stability. The double-plate technique is advantageous in circumferential or atrophic bone loss due to the axial and torsional stable configuration. Furthermore, the bone grafting technique was also a decisive procedure in achieving a satisfactory outcome. We used the 360° autogenous bone graft technique at the site of bone loss. The bone autograft material was acquired from the anterior iliac crest. It not only can supply biomechanical connections at the defect site, but also rebuild the clavicle blood circulation [22]. Similarly, Haj et al. reported that the treatment of nonunion after limb fracture with or without double plate autograft has achieved satisfactory results [27].

**Table 3:** Summarization of clavicle nonunion treated by different treatments

Authors	Type of treatment	Mean Age (year)	Follow-up (month)	No. of patients	Type of nonunion	Results	Time for union (weeks)
Schnetzke et al. [22]	Plate + graft	38.7	8.8 ± 2.4(1) 9.0 ± 3.0(2) (years)	58	19 At, 11Ht	72% union(1) 93.1% union(2)	10.3 ± 9.5(1) 4.7 ± 3.4(2) (months)
Huang et al. [23]	LC-DCP	43.4 ①and 49.3②	20.4	51	51Ht	100% union with LC-DCP	9.3① and 9.1②
Slavko et al. [8]	Ilizarov external Fixator	38.7 ± 12.4	45.4	12	12 At	100% union	10
Kabak et al. [24]	DCP and LC-DCP, graft	39.3 and 43.6	44.2	33	25 At, 8 Ht	100% union with LC-DCP	9.2 (LC-DCP) 11.9 (DCP)
Sadiq et al. [26]	DCP, 2-plates	39	NA	20	NA	100% union	17
Jones et al. [25]	Plate + graft	None	33	14	12 At, 2 Ht	93% union	15.6
Our treatment	2-Plates + graft	35	45.4 ± 12.6	12	7 Ht, 5 At	100% union	9

*LC-DCP* limited contact-dynamic compression plate, *DCP* dynamic compression plate, *Ht* hypertrophic, *At* atrophic, *none* not mentioned

^①^Failure of initial surgical treatment (group 1)

^②^Failure of conservative treatment (group 2)

^(1)^Plate revision without bone graft

^(2)^Plate revision with iliac crest bone graft

In our experience, double-plate combined with autogenous cancellous bone graft technique is necessary for patients with the atrophic clavicular nonunion, especially for bone defects. An auxiliary small plate in front of the bone defect could increase the fixation stability and maintain the graft position at the nonunion site. Double plates could also allow shoulder girdle early rehabilitation exercise postoperatively due to the absolute stability. In patients with atrophic nonunion, large bone defects often existed after removal of the sclerotic bone, so it is necessary to use an autogenous bone graft to restore clavicle length and double plates to achieve rigid fixation. In addition, there are some other key technology in the surgical procedure: (1) the ends of sclerotic bone should be debrided until the observance of the paprika sign, (2) the medullary cavity should be drilled through the K-wire, and (3) the cardinal plate above the clavicle is usually 9 hole AO locking reconstruction plate or LC-DCP plate (3.5 mm, 6 screws, double cortex fixation). (4) Plates should be pre-contoured in terms of the patient’s clavicular anatomy. (5) It is essential to handle 360° autogenous bone graft at a bone defect site. The bone autograft material was acquired from the anterior iliac crest. (6) The auxiliary plate in the anterior-posterior plane is usually 7–9 hole general reconstruction plate or AO LCP(2.7 mm, 4 screws, single cortex fixation). It could improve fixation stability in the anterior-posterior plane of the clavicle.

Data on the demographic characteristics of patients, treatment results, and complication rates were included in our study. However, there are still some limitations to this study. The number of patients was relatively small. Therefore, more patients need to be included in this study to verify the effectiveness of this procedure.

## Conclusion

Our study showed that double-plate with autogenous bone graft may be a prosperous and ideal alternative osteosynthesis technique for treating the atrophic clavicular nonunion, especially those with significant bone gaps. The rigid stability of the double-fixation construct allows the patient to proceed with functional rehabilitation exercise earlier and finally obtain better results. Therefore, double-plate with autogenous bone graft may be a successful salvage procedure when traditional means of achieving union were ineffective.

## Data Availability

The data used to support the findings of this study are included within the article. All data and materials were in full compliance with the journal’s policy.
